# The assessment and management of thermal burn injuries in a UK ambulance service: a clinical audit

**DOI:** 10.29045/14784726.2020.12.5.3.52

**Published:** 2020-12-01

**Authors:** Harriet Ashman, Dean Rigg, Fionna Moore

**Affiliations:** South East Coast Ambulance Service ORCID iD: https://orcid.org/0000-0003-0628-5492; South East Coast Ambulance Service; South East Coast Ambulance Service

**Keywords:** ambulance, burn, management

## Abstract

**Background:**

Although burn emergencies are infrequently encountered, the ambulance service is often the first point of contact for patients in these situations. It is therefore important that these potentially devastating injuries are managed in accordance with the evidence base. Appropriate assessment and management of these patients in the pre-hospital phase will have a significant impact upon their long-term outcomes, such as scarring cosmesis and functionality.

**Aim and objectives:**

This audit was conducted to determine if patients presenting to one UK ambulance service with thermal burn injuries were managed safely, effectively and in a timely manner. Areas highlighted for improvement will assist in directing future pre-hospital research and educational requirements. Epidemiological data will also be provided.

**Results:**

278 thermal burn incidents occurring from June 2017 to May 2018 (inclusive) were included in this audit. A larger proportion of burn patients were paediatrics who fell into the 0–10 age category, most burn patients were injured at a home address and only nine of the overall sample were major burns. Only 35% of patients received adequate cooling of their burns, an essential first aid intervention. The assessment of pain (87%) and provision of analgesia (75%) showed a higher compliance rate. However, only 54% had pain reassessed after analgesia. There was a near 100% compliance rate for patients being managed without hydrogel dressings and topical medicines.

**Conclusion:**

The results indicate several areas for improvement within the ambulance trust. Of importance is the application of basic first aid, such as cooling. It is important not only to improve education among staff but also to understand non-compliance. It should be acknowledged that assessment of pain and provision of analgesia demonstrated far higher compliance compared to current pre-hospital evidence. Several points for education and research have been identified.

## Background

The emergency services are often the first point of contact for patients following thermal injuries. The initial mangement of these patients is key to improving short- and long-term outcomes. Evidence in particular supports the role of cool running water for 20 minutes to reduce time to wound healing, which is directly related to improved cosmetic and functional scar outcomes (Cuttle, Kempf, Kravchuk, Phillips et al., 2008; Cuttle et al., 2010; Venter et al., 2007; Wood et al., 2016). Cooling should only be delayed or omitted in life-threatening circumstances (Battaloglu et al., 2019). Assesment of burn injuries is also required to determine severity and the subsequent interventions required, both pre-hospital and in hospital, such as the need for intravenous (IV) fluids. Cling film as a primary dressing choice is considered appropriate to cover the wound, act as a barrier to infection, provide an analgesic effect and allow continuous wound assessment (Brown et al., 2016; LSEBN, 2018).

In 2016, hydrogel dressings were removed from South East Coast Ambulance service (SECAmb) ambulances, under direction of the London South East Burns Network (LSEBN) (Stiles et al., 2018). Very little evidence exists demonstrating their cooling efficacy. They may retain heat in the absence of air convection, potentially worsening the burn (Goodwin et al., 2015; Cho & Choi, 2017).

Burn injuries are of low incidence in developed countries, and anecdotally burn education in most healthcare settings is limited, which may result in inconsistent and poorly informed practice. Evidence suggests that UK healthcare professionals have limited knowledge and awareness of burns (Tay et al., 2013), reflected also by findings from the Netherlands and Australia (Breederveld et al., 2011; Rea et al., 2005). National and international research addressing pre-hospital education and management of thermal burns suggests poor compliance with basic and key first aid interventions (Breederveld et al., 2011; Fiandeiro et al., 2015; Tay et al., 2013). Analgesia is also of significant importance in this patient group: pre-hospital intervention has been found as a predictor of patients not receiving appropriate analgesia (Baartmans et al., 2016).

Providing the best quality management at the earliest opportunity significantly impacts upon patient outcomes. As pre-hospital staff are often the first point of contact in burn injuries, the findings of the research are concerning. This audit was undertaken within SECAmb to determine whether the Trust was providing optimal care to this patient group, and which areas of burn care are indicated for improvement, ongoing education and research.

## Aim and objectives

The aim of the audit was to ensure that patients presenting to SECAmb with thermal burn injuries are managed safely, effectively and in a timely manner, in line with standards set out by the LSEBN and Joint Royal College Ambulance Liaison Committee (JRCALC) guidelines.

The objectives set out prior to the audit were:

- To ensure that patients with thermal burn injuries are assessed as per JRCALC and LSEBN guidelines.
- To ensure that patients with thermal burn injuries are treated as per JRCALC and LSEBN guidelines.

## Standards and guidelines

Standards for this audit were developed from the UK Ambulance Services Clinical Practice Guidelines 2016, JRCALC (Brown et al., 2016) and guidance from LSEBN (2018) on initial management of burns (Table 1).

**Table 1.** Audit standards.

	Standard	Target	Exception	Reference
1	Patients will receive a minimum of 15–20 minutes of cooling in thermal burns.	100%	Patient refuses, no access to running water, life-threatening concerns that take priority, patient is hypothermic, burn injury is older than 3 hours, there is cooling prior to arrival.	Brown et al. (2016); LSEBN (2018).
2	Patients with thermal burns will be assessed to determine whether they need analgesia.	100%	Patient refuses, unable to assess.	Brown et al. (2016).
3	Patients who are in pain will be offered analgesia.	100%	Unable to assess pain, contra-indications for analgesia, patient refusal, analgesia not required, already given analgesia.	Brown et al. (2016).
4	Pain will be reassessed following analgesia.	100%	Unable to assess pain, patient refusal, analgesia not administered.	Brown et al. (2016).
5	Patients with burns will have percentage total body surface area burned (%TBSAB) documented.	100%	Patient refuses, unable to assess.	Brown et al. (2016); LSEBN (2018).
6	Patients with thermal burns will have depth assessed and documented.	100%	Patient refuses, unable to assess.	Brown et al. (2016); LSEBN (2018).
7	Patients with thermal burns will not be managed using water gel-style burns dressing or other topical medicines.	100%	Patient refuses, bystander application, unknown dressing used.	LSEBN (2018).
8	Burns will be covered using cling film.	100%	Patient refusal, unknown dressing used, bystander application, inappropriate to apply.	Brown et al. (2016); LSEBN (2018).
9	Patients with major thermal burns should receive IV fluids as per JRCALC guidance.	100%	Patient refuses, contra-indicated, not required, unable to cannulate.	Brown et al. (2016).

The 2018 LSEBN guidelines were used as they provide guidance to the locality concerned and have been referenced throughout. Although they were released after the audit was conducted, they are unchanged from the previous version in areas relevant to this audit. The JRCALC guidelines are also used by clinicians to support decision making and therefore direct the standards. Although there are more recent guidelines published in 2019, the 2016 guidelines were in use at the time and therefore informed the standards.

## Sample

The sample included all incidents relating to thermal burns from the selected 12-month period (June 2017 to May 2018 inclusive).

The audit sample was identified using the Trust’s patient clinical record (PCR) database. All completed PCRs are coded to reflect the incident type attended. All PCRs with crew condition codes D14, G03, M05 and M11 were selected (see Table 2). The other discriminators used were PCRs where the burns %TBSAB (total body surface area burns) field or the Superficial, Partial Thickness or Full Thickness were completed.

**Table 2.** Crew condition codes.

Code	Definition
D14	Oral scalding
G03	Burns
M05	Major burns
M11	Electrocution

1390 incidents were identified as the initial sample. These incidents were all reviewed and removed if they met any of the exclusion criteria (Table 3). After exclusion criteria were applied, 278 incidents remained for review and made up the block sample to be analysed.

**Table 3.** Exclusion criteria for burn incidents.

Not burn incidents	1027
Incomplete PCR	4
Illegible PCR	7
Duplicate	1
Hospital transfer (to burns unit)	24
HEMS provided primary care	3
Sun burn only	10
Not acute burn	10
Burns – not of a thermal mechanism	26

## Data source

Data were extracted from PCRs completed by the attending crews. Fields documenting sex, age, pain score, depth of burn, analgesia and %TBSAB were used to provide data. The free-text section was also read to identify any further data.

## Methods

The project team included a paramedic with expert interest in burn care, the Head of Clinical Audit and another paramedic seconded to the clinical audit team. They collectively determined the audit type, the standards for audit (Table 1) and how they were to be met at the initial meeting. The audit is a clinical audit of the assessment and management of thermal burn injuries within SECAmb.

The data were collected retrospectively by the paramedic leading the audit and then analysed using Microsoft Excel. Data were reviewed and validated regularly by two other members of the audit team.

An initial sample of 50 incidents were selected to pilot the methodology and standards proposed. From this it became clear that further exceptions to care standards would need to be included to provide fair representation of patient management. For example, patients receiving bystander cooling were marked as exceptions because the clinicians would not meet the standard, but there was no compromise to care received. Another finding from the pilot was that clinicians were not consistently documenting the time that cooling was carried out for. The audit team decided that if a duration of 15–20 minutes had not been clearly documented then the audit standard would not be met. This was rationalised because cooling of a shorter duration has been evidenced to have limited patient benefit. The initial standard set out for cooling duration was 20 minutes, as per LSEBN and British Burn Association guidelines (Battaloglu et al., 2019); this was altered to 15–20 minutes to reflect JRCALC also.

The final audit report was written by Harriet Ashman, Operational Paramedic. The audit was then presented at the Trust’s Clinical Audit and Quality Sub-Group meeting on 13 August 2019, where it was approved.

## Caveats

A widely accepted limitation of clinical audit using retrospective data is that it is subject to inaccurate documentation: incomplete documentation may provide misleading or incorrect results. For example, if there was no crew condition code provided, these incidents would not have been included. Also, if no cooling was documented it was assumed that it did not occur. It is acknowledged that the compliance rate may have been higher, and that part of this result may be attributable to poor documentation.

It was also noted that this sample may not fully represent burn patients attended by SECAmb; there is a loss of data for patients who received onward management from Helicopter Emergency Medical Services (HEMS). When there is HEMS involvement, care is not provided solely by SECAmb, therefore the results may suggest that fewer major burns are attended.

The limitations of auditing pain management should not be overlooked. Not only is pain a subjective experience that is difficult to assess, but it is also challenging to determine whether analgesia requirements have been met from documentation. Also, the audit does not focus on non-pharmacological methods of pain relief, which are equally important.

## Results

The epidemiology of the selected sample is detailed in Table 4. Patients often had burns of multiple depths, as demonstrated in Table 5.

**Table 4.** Patient demographics.

Parameter	N	(%) of sample
**Age**		
0–10	88	32
11–20	26	9
21–30	29	10
31–40	32	12
41–50	28	10
51–60	18	6
61–70	14	5
71–80	23	8
81–90	13	5
91–100	5	2
Unknown	2	1
**Sex**		
Male	150	54
Female	122	44
Unknown	6	2
**Place of injury**		
Home address	216	78
Workplace	12	4
Public place	33	12
Other	3	1
Unknown	14	5
**Non-thermal burns**		
Chemical	14	Not inc.
Electrical	12	Not inc.

**Table 5.** Burn severity.

	N	%
**Depth**	
Superficial / 1st degree	100	65
Partial / 2nd degree	76	49
Full thickness / 3rd degree	7	5
**TBSAB**		
5% and under	81	61
6–10%	33	25
10–14%	9	7
15–25%	7	5
>25%	2	2

Compliance rates from the audit are displayed in Table 6.

**Table 6.** Compliance with audit standards.

Standard	Compliance	
	N	%
Cooling	61	35
Need for analgesia assessed	240	87
Patients given analgesia	182	75
Reassessed after analgesia	112	54
%TBSAB documented	132	48
Burn depth documented	154	56
Not managed with water gel or topical medicines	256	97
Cling film applied	84	33
IV fluids given	3	33

Exceptions to each standard (Table 1) were agreed prior to the audit process. Where an incident met an exception, it was documented and excluded from the overall compliance.

## Observations

Areas of good practice:

- 87% of patients had pain assessed
- 75% of patients were given analgesia
- 97% of patients were not managed with water gel dressings or any other topical medicines.

Areas for improvement:

- 35% of patients had their injuries cooled with 20 minutes of running water
- 54% of patients had their pain reassessed following analgesia being given
- Cling film was applied in 33% of burns
- 48% of patients had the %TBSAB assessed and documented
- Depth of burn was assessed and documented in 56% of patients
- IV fluids were administered in 33% of major burns.

## Discussion

The large majority of burn patients were children aged 0–10; this finding is consistent with current literature in the UK (Stylianou et al., 2015) and globally (WHO, 2008). This finding presents several implications for practice; paediatric patients, much like those with burn injuries, are less frequently encountered by the ambulance service than adults. This further compounds effective assessment and management (Rutkowska and Skotnicka-Klonowicz, 2015; Seid et al., 2012). They are also more likely to suffer long-term implications of injuries inappropriately managed due to underdeveloped dermal and epidermal layers, resulting in deeper and potentially more debilitating wounds (Stamatas et al., 2010). As children grow and develop, subsequent scar tissue can limit functionality through contractures and have a huge cosmetic impact. In addition, survivors of severe burns are signifcantly younger than non-survivors (Kallinen et al., 2016). Improving the care delivered to all burn patients at the initial point of contact will have a larger cost benefit on the healthcare system as a whole, reducing recovery and rehabilitation time (Griffin et al., 2019; Nguyen et al., 2002), and paediatric patients will receive the largest benefit from this.

The results also show that burn injuries most commonly occur at a home address, which is well documented in the literature (Griffin et al., 2019). This raises questions around such low compliance with appropriate cooling when there is easily available running water. This cannot be answered by this audit alone and will need further research. The cooling of burns reduces time to wound healing, as well as the depth, severity and scarring of the wound (Griffin et al., 2019; Harish et al., 2019; Wright et al., 2019). However, crews were only compliant with cooling for an appropriate duration in 35% of patients. This may have a huge impact on burn survivor outcomes. Current literature suggests a global lack of knowledge and awareness of burn management across parents, carers and healthcare professionals (Graham et al., 2012; Rea et al., 2005; Tay et al., 2013). Although these findings are not specific to the ambulance service, this combined with evidence that pre-hospital burn management has room for improvement (Fein et al., 2014) suggests that a lack of knowledge may contribute to poor compliance within SECAmb also.

High compliance rates were found for assessing the need for and appropriately offering analgesia. This is a very positive finding as it is recognised that burn-related pain is often undertreated in the pre-hospital environment (Baartmans et al., 2016). Burn-related pain is a significant contributor to morbidity, not just affecting functional outcomes but also psychological recovery. Pain treated early in the patient journey is far easier to manage long term (McGhee et al., 2011), therefore maintaining high compliance will have beneficial long-term impacts on burn patients attended by SECAmb.

The assessment of %TBSAB was only documented in 48% of cases and should be addressed. The importance of %TBSAB lies in directing care pathways, and determining the patient’s need for IV resuscitation fluids and the most appropriate destination. There was a similar rate of compliance in depth assessment. While this is also considered an area that may need improving, it should be noted that the significance of pre-hospital depth assessment is underemphasised by burn specialists. Observational assessment is often inaccurate, particularly in the first 48 hours, even when carried out by burn specialists (Malic et al., 2007; Thom, 2017). Because of the dynamic nature of burn wounds, depth progression is common in the early stages. A finding of interest regarding assessment is that crews describing the burn appearance in the free-text section often matched this to an incorrect burn depth, also suggesting a need for education. For example, where burns were described as having blisters they were then documented as superficial; however, isolated erythema is the only presentation of superficial burns. The implication of this in practice is the risk of inappropriate decision making regarding the patient’s ongoing care. There are also only three depth options provided on the PCR at the time of audit (superficial, partial thickness, full thickness) that do not reflect current burn classification (LSEBN, 2018), highlighting a flaw in the documentation platform within the Trust.

Non-compliance with not applying hydrogel dressings and topical medicines was only 3%. This is largely attributable to SECAmb stopping carrying these dressings in 2016. Nevertheless, this finding is very encouraging as the possible detrimental effects of using these dressings in isolation are becoming better understood. Hydrogel (and similar) dressings have limited evidence to support use in the pre-hospital setting (Goodwin et al., 2015) and there is emerging evidence suggesting that they may be detrimental by retaining heat in the wound, extending the damage and subsequent scarring (Cho & Choi, 2017; Cuttle, Kempf, Kravchuk, George et al., 2008).

Application of cling film as a lightweight, transparent and non-adherant dressing was only documented in 33% of incidents. Both JRCALC (Brown et al., 2019) and LSEBN (2018) direct clinicians to this as their primary dressing. Not only does it provide a barrier to infection, but it also helps relieve pain by preventing air movement over exposed nerve endings (Bourke & Dunn, 2015). All front line vehicles are stocked with cling film, therefore compliance can and should be improved. It is recognised that non-specific wording or a lack of documentation may contribute to the rate of non-compliance. For example, some crews documented that they dressed the wound but they were not clear about what was used, such as ‘dressing applied’, and would have been considered as non-compliant.

The final audit standard was administration of IV fluids as per JRCALC guidance. Only nine patients met the criteria for severe burns, >15% TBSAB in adults and >10% TBSAB in children. As noted, this may not be truly reflective because of the removal of HEMS-attended incidents and clinicians not consistently providing an estimation of %TBSAB. Nevertheless, the current literature does reflect this finding, showing a far higher incidence of minor burns nationally (Stylianou et al., 2015). Of this cohort, only 33% received the necessary IV fluids. Hypovalemic shock, organ hypoperfusion, cardiac dysfunction and wound progression may result from under resuscitation of fluids in a large burn injury (Bacomo & Chung, 2011). There were also no documented exceptions for patients not receiving IV fluids, such as inability to cannulate, so it is not clear why this standard was not met. This finding requires attention to ensure patients with severe burns receive the correct package of potentially life-saving treatment.

It must not be overlooked that burns are infrequently encountered by ambulance crews, and as with any area of medicine it is more challenging to manage presentation where there has been little exposure. Also, the injury or scene presented is often distressing, which can confound adherence with best practice. This audit is a constructive project and does not seek to overlook the hard work of dedicated and caring individuals.

## Recommendations

This audit identified that several elements of best practice in relation to thermal burns were not met or documented appropriately.

To better understand the reasons for this, investigation and research could be undertaken to help direct improvement strategies. Qualitative interviews conducted with clinicians may provide valuable insight into current issues and direct specific improvement measures. The process of conducting such research may be lengthy; while this is developed, more urgent changes need to be addressed to reduce the impact of poor compliance on patients’ long-term outcomes.

It has been highlighted by the audit that burns are an infrequently encountered presentation, particularly in the paediatric population. The evidence base also highlights an overall lack of burn knowledge across healthcare professions. A lack of exposure to such incidents may impact upon confidence, assessment and management. While it is not possible to increase exposure, it would be practical to implement further educational packages across the Trust, to raise awareness of the issues highlighted and improve staff knowledge and confidence. This could be implemented through voluntary CPD sessions, e-learning and mandatory training. There is evidence that staff receiving further burn care education showed improved clinical performance (Breederveld et al., 2011).

Another concern raised in the audit was the consistency of clear documentation; for instance, the lack of clarity provided regarding duration of cooling or specific dressings used. While highlighting the importance of these interventions and documentation to staff may help improve compliance, adapting the platform used for documentation may also help. Another flaw identified in the PCRs was the burn classification field, which requires updating to reflect current national standards. Deep dermal and mixed depth should be included in the depth fields, alongside the current depths (superficial, superficial-partial thickness and full thickness) to fully reflect the diversity of burn depth and match the terminology and categorisations currently used by burn services (LSEBN, 2018).

The audit should be conducted again, once changes have been made with enough time for adjustment, to show any improvement.

## Learning points

The below points were identified through the audit process and should be reconsidered before a re-audit is undertaken:

- The audit process used identified many non-burn-related incidents, reducing the timeliness of the audit process. This is likely to be improved by the introduction of electronic PCR and auditing systems.
- Timeliness did not adhere to expectations; deadlines and workload should be reconsidered for future audit.
- Crew condition codes and guidelines were changed once the audit was approved, so care should be taken that these elements are updated on re-audit.

## Acknowledgements

We thank the Trust’s clinical audit team for their support throughout*, *particularly Eleanor Jaquet.

## Author contributions

HA collected audit data and compiled the report. DR directed the audit process, regularly validated the data and provided critical revisions to the report. FM provided critical revisions to the report and approved request for publication. HA acts as the guarantor for this article.

## Conflict of interest

None declared.

## Ethics

Not required.

## Funding

None.
